# Carbocisteine Improves Histone Deacetylase 2 Deacetylation Activity via Regulating Sumoylation of Histone Deacetylase 2 in Human Tracheobronchial Epithelial Cells

**DOI:** 10.3389/fphar.2019.00166

**Published:** 2019-02-27

**Authors:** Yun Song, Dan-Yi Chi, Ping Yu, Juan-Juan Lu, Jian-Rong Xu, Pan-Pan Tan, Bin Wang, Yong-Yao Cui, Hong-Zhuan Chen

**Affiliations:** ^1^Department of Pharmacy, Huashan Hospital, Fudan University, Shanghai, China; ^2^Department of Pharmacology, Shanghai Jiao Tong University School of Medicine, Shanghai, China; ^3^Puyang Health School, Puyang, China

**Keywords:** small ubiquitin-related modifier, histone deacetylase 2, cigarette smoke extract, carbocisteine, GSH/thiol

## Abstract

Histone deacetylase (HDAC) 2 plays a vital role in modifying histones to mediate inflammatory responses, while HDAC2 itself is commonly regulated by post-translational modifications. Small ubiquitin-related modifier (SUMO), as an important PTM factor, is involved in the regulation of multiple protein functions. Our previous studies have shown that carbocisteine (S-CMC) reversed cigarette smoke extract (CSE)-induced down-regulation of HDAC2 expression/activity in a thiol/GSH-dependent manner and enhanced sensitivity of steroid therapy. However, the mechanism by which S-CMC regulates HDAC2 is worth further exploring. Our study aimed to investigate the relationships between HDAC2 sumoylation and its deacetylase activity under oxidative stress and the molecular mechanism of S-CMC to regulate HDAC2 activity that mediates inflammatory responses in human bronchial epithelial cells. We found that modification of HDAC2 by SUMO1 and SUMO2/3 occurred in 16HBE cells under physiological conditions, and CSE induced SUMO1 modification of HDAC2 in a dose and time-dependent manner. K462 and K51 of HDAC2 were the two major modification sites of SUMO1, and the K51 site mediated deacetylation activity and function of HDAC2 on histone H4 that regulates IL-8 secretion. S-CMC inhibited CSE-induced SUMO1 modification of HDAC2 in the presence of thiol/GSH, increased HDAC activity, and decreased IL-8 expression. Our study may provide novel mechanistic explanation of S-CMC to ameliorate steroid sensitivity treatment in chronic obstructive pulmonary disease.

## Graphical Abstract

Carbocisteine improves HDAC2 deacetylation activity via regulating HDAC2 sumoylation in human tracheobronchial epithelial cells.

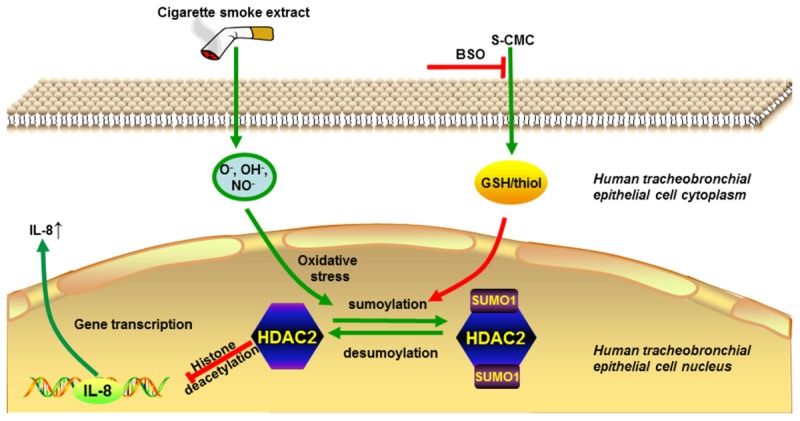

## Introduction

Histone deacetylase (HDAC) 2 plays a crucial role in modifying histones to regulate the expression of inflammatory genes (Barnes, 2005, 2009; Ito et al., 2005). Our previous studies have shown that carbocisteine (S-CMC), an anti-oxidant agent extensively used as adjunctive therapy in the treatment of chronic obstructive pulmonary disease (COPD), could reverse the down-regulation of HDAC2 induced by oxidative stress and enhance the sensitivity of human alveolar epithelial cells to glucocorticoid (GC) treatment (Song et al., 2015). However, it remains to be understood regarding the mechanism by which S-CMC ameliorates HDAC2 expression and/or activity.

Protein expression/activity is largely dependent on epigenetic modulations or post-translational modification (PTM) such as phosphorylation, nitrosylation, ubiquitination and sumoylation. PTM indicates that the proteins encoded by genes are further modified by a series of enzymatic reactions, resulting in the specific chemical modification of the amino acid residues on the protein peptide chain, which usually involves chemical modifications of proteins via linkage of chemical groups, lipids, sugars or polypeptides with the proteins (Flotho and Melchior, 2013; Woodsmith et al., 2013). As a result, these chemical modifications cause a variety of structural and functional alteration that mediate biological and/or pathological process (Banks et al., 2000; Mann and Jensen, 2003; Segré and Chiocca, 2011; Woodsmith et al., 2013; Lee and Kim, 2015).

HDAC2, as a regulating protein of histone acetylation, is also modified by post-translational regulation (Brandl et al., 2009; Segré and Chiocca, 2011; Eom and Kook, 2014). Studies have shown that HDAC2 expression/activity inhibited by oxidative stress is closely associated with the epigenetic modifications, such as phosphorylation (Adenuga et al., 2009), nitrosylation (Nott et al., 2008; Osoata et al., 2009; Malhotra et al., 2011), ubiquitination (Adenuga et al., 2009) and so on. SUMO, as an important PTM factor, is involved in the regulation of HDAC2 (Brandl et al., 2012; Wagner et al., 2015).

SUMO, a small ubiquitin-like modifier, is widely expressed in eukaryotic cells and is composed of highly conserved four family members, including SUMO1, SUMO2, SUMO3, and SUMO4. SUMO covalently binds to a lysine residue of substrates. Sumoylaton is a dynamic reversible process and occurs under physiological or pathological condition (Johnson, 2004; Flotho and Melchior, 2013). It plays an important role in regulating the function and stability of proteins (Johnson, 2004; Lomelí and Vázquez, 2011). For example, sumoylation of HDAC2 promotes NF-KappaB-dependent gene expression (Wagner et al., 2015). Sumoylation of HDAC2 at lysine 462 permits HDAC2 binding to p53, which in turn blocks the recruitment of p53 into the promoter regions of targeted genes that mediates cell cycle and cell apoptosis (Brandl et al., 2012). Protein sumoylation is sensitive to diverse cellular stresses, and oxidative stress not only directly modifies proteins but also lead to indirect changes in SUMO modifications that regulate protein functions (Bossis and Melchior, 2006). However, the relationships between oxidative stress induced by cigarette and SUMO modification of HDAC2 has not been reported so far, which may further reveal the mechanism of S-CMC to regulate HDAC2.

In this study, we aimed to investigate the effects of S-CMC on cigarette smoke extract (CSE)-induced HDAC2 SUMOylation and the subsequentinflammatory responses. We first determined the SUMO modification of HDAC2 under a physiological condition, and explored the effects of oxidative stress induced by cigarette on HDAC2 sumoylation. Next, we determined the specific modification site of HDAC2 and revealed the effects of SUMO modification on the biological function of HDAC2. Finally, we investigated the effects and mechanisms of thiol antioxidant S-CMC on the HDAC2 sumoylation, to provide new targets for the treatment of steroid-resistant COPD.

## Materials and Methods

### Cell Culture and Treatments

The human tracheobronchial epithelial (16HBE) cell line [obtained from the American Type Culture Collection (Manassas, VA, United States)] was maintained in DMEM medium supplemented with 10% fetal bovine serum, L-glutamine (2 mM), penicillin (100 U/mL), and streptomycin (100 U/mL). Unless otherwise stated, cell culture reagents were purchased from Gibco (United States). CSE was prepared as previously reported (Song et al., 2015). Briefly, CSE was prepared by the combustion of one cigarette (12 mg tar/cigarette; Double Happiness, China), using a pump and passing the smoke through 10 mL of non-FBS culture medium at a rate of 5 min/cigarette. The resulting solution was adjusted to pH 7.4 with 1.0 M NaOH and strained through 0.22 μm gauge filters. The obtained solution represented 100% strength and was diluted to the desired concentration with culture medium. The fresh CSE was used within 30 min.

Cells were pre-incubated with or without S-CMC (Sigma-Aldrich, United States) and buthioninesulfoximine (BSO; Sigma-Aldrich, United States) for 1 h and then exposed to CSE. These treatments showed no significant cytotoxic effects as measured with a cck-8 counting kit (Dojindo, Japan) and trypan blue exclusion method.

### Immunoprecipitation

16HBE cells were plated in 10 cm cell culture dish, washed with ice-cold PBS and scraped the dish with 600 μL ice-cold lysis-buffer (20 mM Tris-HCl pH 7.4, 150 mM NaCl, 10%glycerol, 0.3%Triton X-100, 0.5%NP-40, 4 mM EDTA) supplemented with 1mM NaVO3, 1mM PMSF, 20mM N-Ethylmaleimide (Sigma, prepared fresh) and Complete protease inhibitor cocktail (Roche, 1:100) and pipetted into 1.5 mL Eppendorf tubes. Cells were incubated on ice for 30 min and sonicated in an ice water bath (amplitude 20%, 3 × 5 s), then centrifuge 14,000 g for 5 min at 4°C, gently transfer the supernatant to a new tube. BCA kit was used to measure the total protein of each tube, and the total protein concentration of each tube was adjusted to the same level with IP lysate. Two hundred micro liter l lysates were enriched by adding anti-HDAC2 or SUMO antibodies (1–2 μg) overnight at 4°C, then added 20 μL pre-cleared Protein A/G-agarose (CST) for 6 h. Complexes were centrifuged at 13,000 × g at 4°C for 30 s. The pellet were washed 5 times with lysis buffer and resuspended in 30 μL 2 × loading buffer (+100:1 PMSF) and heated to 100°C for 5 min. The samples were centrifuged at 2000 rpm, and the supernatant was subjected to protein separation by 10% SDS-PAGE. Western blot analysis was performed as described below.

### Western Blot

For the analysis of proteins expression, the 16HBE cells lysate was prepared by RIPA Lysis Buffer (+PMSF, 100:1; Beyotime Institute of Biotechnology, China). The total protein was extracted by centrifugation (14,000 × *g*, 4°C, 10 min) in the supernatant for gel electrophoresis. After western transfer, PVDF membranes were probed with mouse anti-HDAC2 antibody, followed by horseradish peroxidase-conjugated anti-mouse (1:5000) antiserum (Santa Cruz Biotech, United States). Protein bands were visualized using SuperSignal (Pierce). The membranes were lastly re-probed with an anti-IgG antibody (Signalway Antibody, United States) as loading controls. The quantitiy of loading samples were adjusted to ensure the same contents of HDAC2 in each group according to the initial result detected by IP-WB.

### Immunofluorescence

Cells were grown on chamber slides and treated as designed. After intermediate washes with cold phosphate buffered saline (PBS), cells were fixed with 4.0% paraformaldehyde in PBS for 15 min at RT. The cells were rinsed in cold PBS and blocked in 5% bovine serum albumin for 1 h at RT. Cells were then incubated with primary antibodies, HDAC2 (1:200 dilution; CST), SUMO1 (1:200 dilution, CST) and SUMO2/3 (1:200 dilution, CST) overnight at 4°C, washed with cold PBS, incubated with Alexa Fluor-conjugated secondary antibodies at RT for 1 h, washed with PBS again, and then stained with 1 μg/mL (w/v) 4,6-diamidino-2-phenylindole (DAPI) for 5 min at RT. After washing, images were collected using an Axioscope microscope system (Leica, Germany) at 40× magnifications.

### High Content Screening (HCS) Assay

HDAC2 expression in 16HBE cells were measured by HCS technology based on immunocytofluorescence. Anti-HDAC2 primary antibody (mouse anti-human; Cell Signaling Technology, United States) was incubated overnight, followed by incubation with secondary antibodies (DyLight 555 nm, goat anti-mouse; Cell Signaling Technology, United States) for 60 min in the dark and stained with 4,6-diamidino-2-phenylindole (DAPI, Sigma-Aldrich, United States). Fluorescence was detected using a HCS Reader (Thermo Fisher Scientific, United States) at 10× magnifications to quantify HDAC2 expression.

### Short Hairpin RNA (shRNA) Interference

HDAC2 site-specific mutagenesis was performed by transfecting 16HBE cells with HDAC2 shRNA (Shanghai Gima Co., Ltd., China) according to the manufacturer’s instructions. 16HBE cells grown to 30–50% confluency were transfected by lentiviral-delivered shRNA (multiplicity of infection = 20), strengthened by polybrene (5 g/mL) for 24 h. Then, the medium was replaced with fresh medium without shRNA and polybrene, and the culture was incubated for another 48 h. The infection efficiency was ≥ 50% according to green fluorescent protein analysis. Cells then were screened with puromycin (2.5 g/mL) for 3–5 days and monoclonal cells were maintained for further experimentation.

### Chromatin Immunoprecipitation (ChIP)- PCR Assays

The ChIP assay was performed using the SimpleChip Enzymatic Chromatin IP kit (Cell Signaling Technology, United States) following the manufacturer’s instructions. The eluted DNA was checked for 100–300 bp fragment enrichment by 8% polyacrylamide gelelectrophoresis. The immunoprecipitated DNA was used amplified using specific gene (IL-8 for humans) promoter qPCR assays. The following PCR primer sequences were used for the IL-8 promoter: forward, 5′-GGGTGCATAAGTTCTCTAG-3′ (position: 3-21) and reverse, 5′-TTCCTTCCGGTGGTTTCTTC-3′ (position: 114-133).

### Statistical Analysis

Quantitative data are presented as means ± standard error of the mean (SEM). Statistical analysis was performed using a one-way analysis of variance followed by the Dunnett’s test for multiple comparisons. All analyses were performed using Prism GraphPad5.0 statistical software. A probability (*p*) value < 0.05 was considered statistically significant.

## Results

### HDAC2 Is Covalently Modified by SUMO1 and SUMO2/3 Under Physiological Condition

**FIGURE 1:**
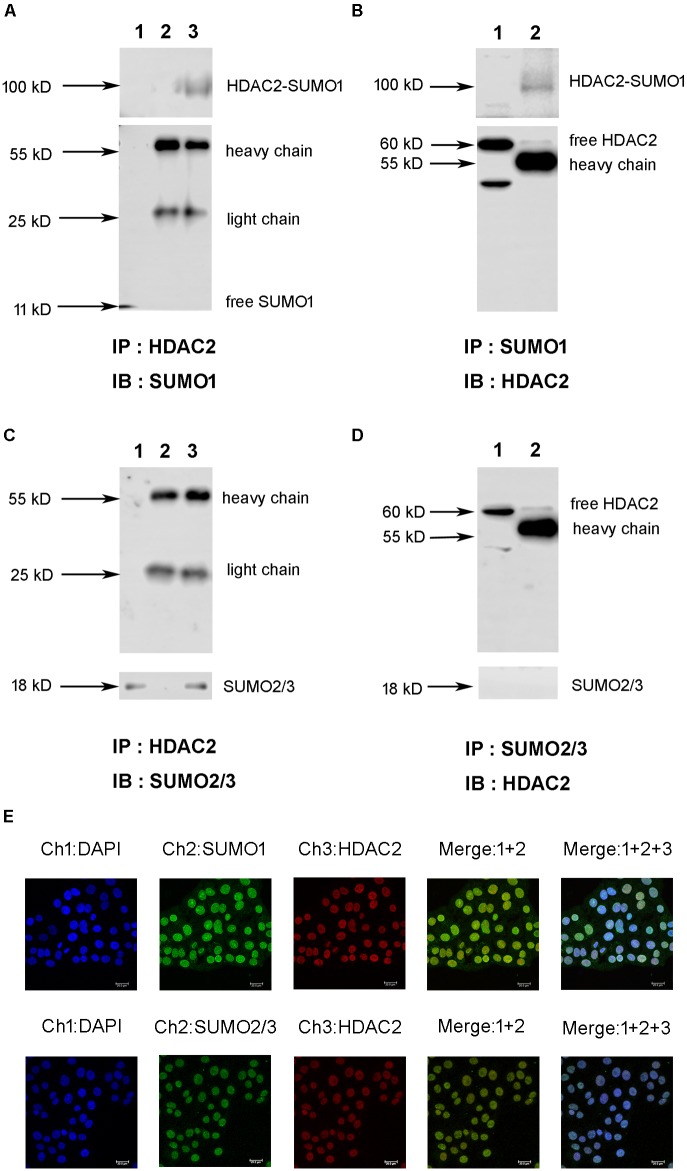
The interaction and colocalization of HDAC2 and SUMO1 or SUMO2/3 in 16HBE cells under physiological condition by immunoprecipitation and confocal microscopy. **(A,C)** Cell lysates were subjected to immunoprecipation using anti-HDAC2 antibodies. SUMOylated HDAC2 was detected by Western blot using anti-SUMO1 or SUMO2/3 antibodies. Lane 1: input (whole cell lysate without IP treatment); Lane 2: immunoprecipitation with IgG (H + L chains) but without HDAC2 antibody; Lane 3: normal IP. **(B,D)** Cell lysates were subjected to immunoprecipation using anti-SUMO1 or SUMO2/3 antibodies. SUMOylated HDAC2 was detected by Western blot using anti-HDAC2 antibodies. Lane 1: immunoprecipitation with IgG (H + L chains) but without SUMO1 or SUMO2/3 antibody; Lane 2: normal IP **(E)** Representative confocal images of fluorescently labeled nucleus (blue, channel 1: 386 nm), SUMO1 or SUMO2/3 (green, channel 2: 488 nm) and HDAC2 (red, channel 3: 555 nm) in 16HBE cells in physiological conditions. There is a colocalization of HDAC2 and SUMOs in nucleus of 16HBE cells (Merge1+2+3).

First, we explored the interaction between HDAC2 and SUMO1 or SUMO2/3 under a physiological condition using immunoprecipitation and immunofluorescent staining. Cell lysates of normal 16HBE cells were subjected to immunoprecipitation using an antibody against HDAC2, followed by western blotting using an antibody against SUMO1. Results exhibited that there was a single ∼100 kD and detected by SUMO1 antibody without free SUMO1 band (∼11 kD), indicating the possible covalent binding of HDAC2 and SUMO1 (Figure 1A). Moreover, the same band at ∼100 kD was observed by reverse immunoprecipitation experiment (Figure 1B), suggesting that HDAC2 is covalently modified by SUMO1 under a physiological condition. Then, the similar IP and IB experiment was used to confirm the interaction between HDAC2 and SUMO2/3. Interestingly, we found only a free SUMO2/3 band (∼11 kD), with no any band that represented the complex of HDAC2 and SUMO2/3 (Figure 1C,D), suggesting the non-covalent interaction may occur between SUMO2/3 and HDAC2.

Next, we sought to visualize if HDAC2 and SUMO1, 2, 3 are co-localized in the nucleus. Immunofluorescent staining showed the colocalization of HDAC2 with SUMO1 or SUMO2/3 in the nuclei of 16HBE cells (Figure 1E). Overlap of HDAC2 with SUMO1 or SUMO2/3 calculated by Image Pro Plus was 86 and 83%, respectively, which also suggested that there might be interaction between HDAC2 and SUMO1 or SUMO2/3 under physiological condition.

### Effects of Cigarette Smoke Extract (CSE) on the Interaction Between HDAC2 and SUMO1

**FIGURE 2:**
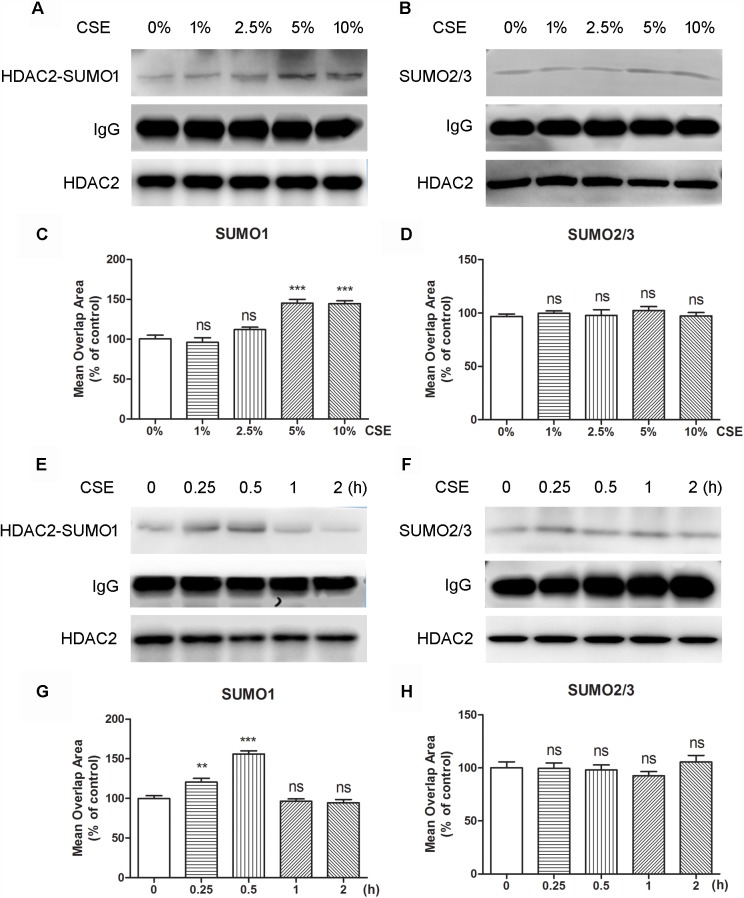
The effects of CSE in different concentrations and different incubation time on HDAC2 and SUMO1 or SUMO2/3 interaction in 16HBE by immunoprecipitation and HCS analysis. **(A,B)** Effects of treatment with CSE under 0, 1, 2.5, 5, and 10% concentrations for 30 min in SUMOylated HDAC2 in 16HBE cells by immunoprecipitation. **(C,D)** Effects of treatment with CSE under 0, 1, 2.5, 5, and 10% concentrations for 30 min in SUMOylated HDAC2 in 16HBE cells by HCS analysis. **(E,F)** Effects of treatment with CSE for 0, 15, 30 min, 1 and 2 h in SUMOylated HDAC2 in 16HBE cells by immunoprecipitation. **(G,H)** Effects of treatment with CSE for 0, 15, 30 min, 1 and 2 h in SUMOylated HDAC2 in 16HBE cells by HCS analysis. Data are expressed as the means ± SEM, *n* = 6. Ns means no significant difference and ^∗∗^*p* < 0.01 and ^∗∗∗^*p* < 0.001 compared to control group (0% CSE or 0 h) using one-way ANOVA with Dunnett *t*-test for selected pairs.

To evaluate if oxidative stress can influence the interaction of HDAC2 with SUMO1 or SUMO2/3, cells were treated with CSE. The immunoprecipitation analysis revealed that the interaction of HDAC2 with SUMO1 was enhanced by CSE in a dose-dependent manner compared to control group and significant difference was found at the concentration of 5% or 10% CSE (Figure 2A). However, no effects of CSE on HDAC2-SUMO2/3 were observed (Figure 2B). Consistent with the results of immunoprecipitation analysis, HCS analysis demonstrated that both 5 and 10% CSE treatment resulted in a significant increase in the interaction between HDAC2 and SUMO1 (Figure 2C), but did not affect the interaction of HDAC2 with SUMO2/3 (Figure 2D). The time course study also showed that exposure of the cells with CSE increased HDAC2-SUMO1 interaction during 0.25–0.5 h (Figure 2E). As expected, the similar results were obtained using the HCS analysis (Figure 2G). The interaction between HDAC2 and SUMO2/3 was, however, not influenced by CSE (Figure 2F,H). These data indicate that CSE induces association between HDAC2 and SUMO1 in a dose- and time-dependent fashion.

### K51 and K462 Are HDAC2 Modification Sites

As is known to us, the molecular weight of SUMO is about 11 kD, but SUMOs appear larger on SDS-PAGE and add ∼20 kD to the apparent molecular weight of most substrates (Johnson, 2004). Based on the founding of the molecular weight of HDAC2-SUMO1 complex, we speculated that there are two SUMO1 proteins covalently attached on the HDAC2. Since SUMO-1 is thought not to form dimer or polymers as well as SUMO2/3 (Tatham et al., 2001), we speculated that there are two different sites of HDAC2 for SUMO1 modification. A few of lysine residues in the sequence of HDAC2 are predicted as the potential sumoylation sites of HDAC2 by three softwares (SUMOplot, SUMOsp2.0, and seeSUMO) based on different prediction algorithm. Results showed that lysine residues K462, K51, K145, and K451 might be the most potential sumoylation sites of HDAC2.

**FIGURE 3:**
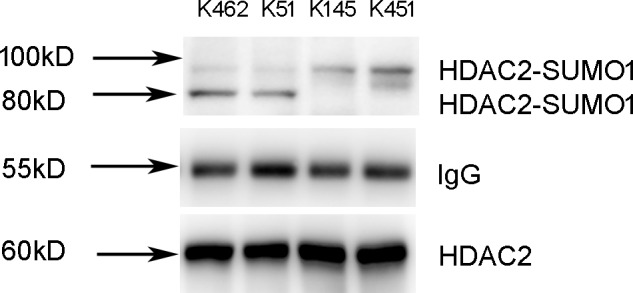
Identification of K51 and K462 as the HDAC2 SUMOylation sites. 16HBE cells were transfected with HDAC2-K462R, HDAC2-K51R, HDAC2-K145R, HDAC2-K451R using lentivirus vector. Cells lysates were subjected to immunoprecipitation using HDAC2 antibody. SUMOylated HDAC2 was detected by Western blot using anti-SUMO1 antibody.

To identify the sumoylation sites of HDAC2, 16HBE cells were transfected with HDAC2-K462R, HDAC2-K51R, HDAC2-K145R, and HDAC2-K451R using lentivirus vector for site-directed mutagenesis. Cells lysates were subjected to immunoprecipitation using HDAC2 antibody. We found that K462R and K51R mutations resulted in the changes of sumoylated HDAC2-SUMO1 complex from 100 to 80 kD, while K145R and K451R mutations did not affect the sumoylation (Figure 3). Therefore, K462 and K51 sites were identified as the possibly primary HDAC2 sumoylation sites.

### Biological Function of SUMO1 Modification of HDAC2

**FIGURE 4:**
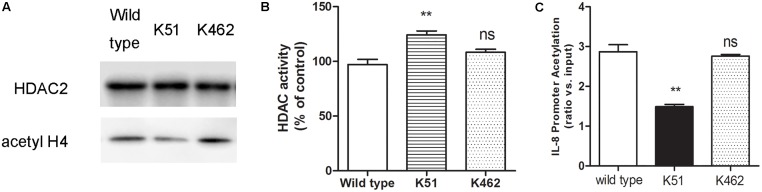
The biological function of SUMO1 modification of HDAC2. **(A)** HDAC2 binding and histone acetylation were analyzed by ChIP analysis in 16HBE wild type, 16HBE-K51R and 16HBE-K462R cells after CSE incubation for 0.5 h. **(B)** HDAC activity in 16HBE wild type, 16HBE-K51R and 16HBE-K462R cells after CSE incubation for 0.5 h. **(C)** histone acetylation of the IL-8 gene promoter in 16HBE wild type, 16HBE-K51R and 16HBE-K462R cells after CSE incubation for 0.5 h. Data are presented as means ± SEM (*n* = 6). Ns means no significant difference and ^∗∗^*p* < 0.01 compared to wild type using one-way ANOVA with Dunnett *t*-test for selected pairs.

Since CSE can induce HDAC2 sumoylation, we reasoned that increased HDAC2 sumoylation leads to suppression of histone deacetylation, while sumoylation-mutated HDAC2 increases deacetylation of histone. To test this hypothesis, we found that 5% CSE significantly increased the acetylation level of histone H4 in wild-type 16HBE cells (Figure 4A), decreased HDAC2 activity (Figure 4B) and increased IL-8 promoter acetylation (Figure 4C) compared with control group by using chromatin immunoprecipitation and HDAC activity assay kit. Compared with the wild type 16HBE cells, K51 mutation resulted in decreased histone H4 acetylation, increased HDAC2 activity and decreased IL-8 acetylation while K462R mutation had no influence in the presence of CSE. These results suggest that CSE enhances SUMO1 modification of HDAC2 at K51 and then regulates histone and IL-8 promoter acetylation.

### S-CMC Reversed the Effects of CSE on HDAC2-SUMO1 Interaction, HDAC Activity and IL-8 Release

**FIGURE 5:**
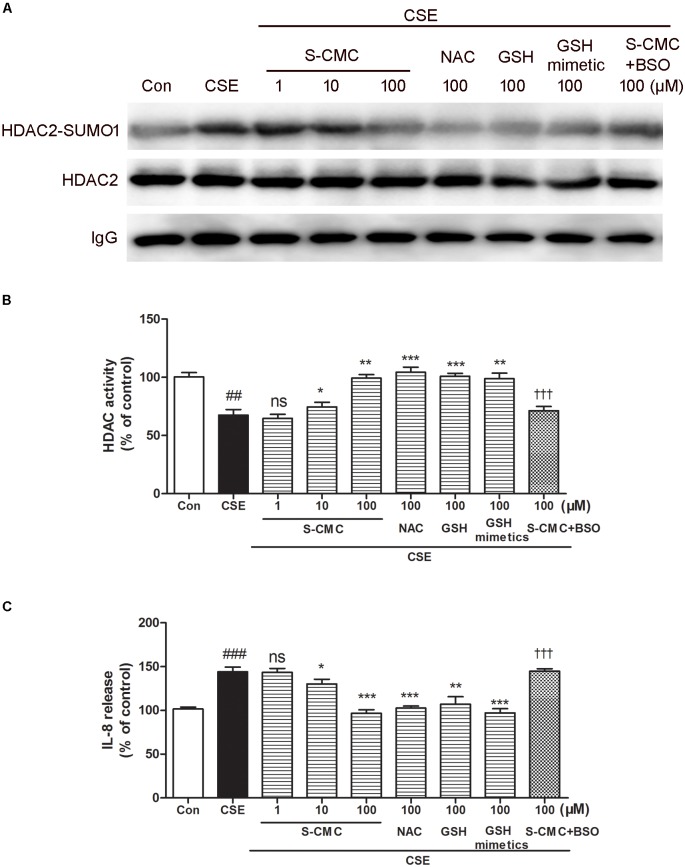
S-CMC reversed the effects of CSE on HDAC2-SUMO1 interaction, HDAC activity and IL-8 release. 16HBE cells were pre-incubated with S-CMC (10^−6^–10^−4^ M) or NAC, GSH, GSH mimetics (10^−4^ M) with or withnot BSO (10^−4^ M) for 1 h, following 5% CSE incubation for 30 min **(A)** Analysis for interaction between HDAC2 and SUMO1 by immunoprecipitation. **(B)** The activity of HDAC in 16HBE cells was detected using a HDAC activity assay kit. **(C)** Level of IL-8 in the indicated groups were measured by ELISA. Data are expressed as the means ± SEM, *n* = 6. ^##^*p* < 0.01 and ^###^*p* < 0.001 compared to control group using unpaired *t*-test. Ns means no significant difference, ^∗^*p* < 0.05, ^∗∗^*p* < 0.01, and ^∗∗∗^*p* < 0.001 compared to CSE group using one-way ANOVA with Dunnett *t*-test for selected pairs. ^†††^*p* < 0.001 compared to the CSE+S-CMC (10^−4^ M) treatment group using unpaired *t*-test.

To investigate whether S-CMC can reverse the effects of CSE on HDAC2-SUMO1 interaction, immunoprecipitation was performed (Figure 5A). It was found that S-CMC could inhibit the CSE-induced SUMO1 modification of HDAC2 in a dose-dependent manner (10^−6^–10^−4^ M). At the same time, other thiol donor drugs (NAC, GSH, GSH mimetics, at the 10^−4^ M) showed similar effects compared with S-CMC (10^−4^ M) (Figure 5A). However, the effect of S-CMC was significantly reversed by the addition of buthioninesulfoximine (BSO), a specific inhibitor of GSH synthesis (Figure 5A). Simultaneously, we also found S-CMC could reverse CSE- induced decrease of HDAC activity (Figure 5B) and increased of IL-8 levels (Figure 5C) in a dose-dependent manner. Other thiol donors (10^−4^ M) also showed similar effects, which was also blocked by the addition of BSO.

## Discussion

Recent studies have revealed that SUMO modification plays an important role in the functional regulation of multiple proteins, such as androgen receptor (Bahnassy et al., 2017), NF-kB pathway (Huang et al., 2003), p53 (Brandl et al., 2012), and HDAC (David et al., 2002; Citro et al., 2013; Wagner et al., 2015). SUMO modification usually occurs on the ΨKXE sequence of the target protein (Johnson, 2004). At present, SUMOsp2.0, seeSUMO and SUMOplot are mainly used to predict the SUMO-modified sites (Teng et al., 2012). Among these predictive softwares, SUMOsp2.0 shows a better recognition of the amino acid sequences of non-ΨKXE sequences (Xue et al., 2006), SeeSUMO software mainly emphasis on the published literature for the prediction of the site (Mei et al., 2017), while SUMOplot matches up the amino acid sequence to predict possible SUMO sites (Yang et al., 2006). Therefore, in order to enhance the reliability of the predictive results, we used all three software programs to predict the SUMO modification site of HDAC2, and combined the three-dimensional structure of the protein to exclude the effects of steric hindrance. As a result, the K462, K51, K145, and K451 amino acid sites of HDAC2 were identified as the potential SUMO modification sites. Our study showed that mutation of K462 and K51, rather than K145 and K451, decreased SUMO1 modification of HDAC2. Therefore, K462 and K51 serve as SUMO1 modification sites of HDAC2. It is noteworthy that K51 is located in the enzyme domain (9–322) of HDAC2, and K462 is located outside this domain. Consistent with our results, other experimental evidence has also demonstrated that K462 site of HDAC2 is a SUMO1 modification site. Interestingly, there are also reports indicating that K462 and K481 site were identified as SUMO2/3 modification sites (Wagner et al., 2017). In our study, it remains to be determined whether K462 is also a modification site for SUMO2/3.

Furthermore, the biological function of SUMO1 modification of HDAC2 at the K51 or K462 sites was also investigated by chromatin immunoprecipitation. Our study showed that K51 site mediated the deacetylation of histones and regulated the transcription of the inflammatory factor, such as IL-8. In contrast to K51, K462 site mutation failed to block HDAC2 function of histone deacetylation. As a result, we speculated that the SUMO1 modification of K462 site might be associated with other protein function. As previously study reported by Brandl et al. showed that SUMO1 modification at the K462 site of HDAC2 mediated the deacetylation of p53 protein, leading to the inhibition of p53 function (Brandl et al., 2012).

Oxidative stress-induced epigenetic modification of HDAC results in the reduction of HDAC2 expression or activity. For example, cigarette smoke can cause phosphorylation of serine residues at 394, 411, and 422, thereby reducing HDAC2 expression and activity (Adenuga et al., 2009). Likewise, oxidative stress induces nitrosylation of tyrosine residue at 253, promoting protease-mediated degradation of HDAC2 (Osoata et al., 2009). Moreover, NO mediates nitrification of cysteine residues at 262 and 274, leading to increases in the transcription of neurotrophic factors-related genes (Nott et al., 2008). In line with these evidence, we found that CSE stimulated HDAC2 modification by SUMO1, not SUMO2/3 in the nucleus, and inhibited HDAC2 activity.

Our previous study has found that oxidative stress induced higher levels of IL-8 and TNF-α (Song et al., 2015), which resisted steroid therapy in COPD. The failure of steroid therapy was improved by the addition of S-CMC in the presence of thiol/GSH (Song et al., 2015). Consistent with these data, we found that S-CMC inhibited CSE-induced SUMO1 modification of HDAC2in a dose-dependent manner, but BSO reversed the inhibitory effects, highlighting that S-CMC-induced HDAC2 activity is dependent on thiol/GSH. Interestingly, other thiol donor drugs had similar effects compared with S-CMC, indicating that thiol donor drugs had the regulatory effect on CSE-induced HDAC2 SUMOylation. Indeed, S-CMC, a cysteine derivative, was reported to enhance the thiol/GSH secretion from human respiratory cells (Guizzardi et al., 2006). Recent studies also confirmed that the presence of thiol/GSH could reverse the deacetylase activity of HDAC2 (Malhotra et al., 2011). Moreover, our previous study revealed that increased thiol/GSH levels by S-CMC were favorable for restoring HDAC2 activity (Song et al., 2015), supporting that thiol/GSH plays a crucial role in reduced SUMO1 modification of HDAC2. Therefore, in consistent with earlier evidence, our studies have provided the molecular mechanisms by which S-CMC blocks CSE-mediated SUMO1 modification of HDAC2 and increases HDAC2 activity in a thiol/GSH-dependent manner. Consequently, HDAC2 induces histone and IL-8 promoter deacetylation and inhibits the expression of IL-8.

## Conclusion

In conclusion, our results show that HDAC2 interacts with SUMO1 under physiological conditions. SUMO1 modification sites of HDAC2 are the K51 and K462 sites, and the SUMO1 modification of the K51 site plays an important role in the deacetylation function of HDAC2. Oxidative stress induced by CSE enhances the SUMO modification of HDAC2. S-CMC or other thiol donor drug treatment can attenuate the CSE- triggered SUMO modification of HDAC2 in thiol/GSH-dependent manner, which provides a mechanistic explanation for the reversal of glucocorticoid resistance by S-CMC in COPD.

## Data Availability

All datasets generated for this study are included in the manuscript and/or the supplementary files.

## Author Contributions

YS, P-PT, and Y-YC participated in research design. J-RX performed the software prediction. YS, J-JL, and PY conducted the experiments. D-YC and BW performed the data analysis and figure edition. YS, Y-YC, and H-ZC wrote or contributed to the writing of the manuscript.

## Conflict of Interest Statement

The authors declare that the research was conducted in the absence of any commercial or financial relationships that could be construed as a potential conflict of interest.

## Abbreviations

BSO: buthioninesulfoximine
ChIP: chromatin immunoprecipitation
COPD: chronic obstructive pulmonary disease
CSE: cigarette smoke extract
GC: glucocorticoid
HCS: high content screening
HDAC: histone deacetylase
IL-8: interleukin
IP: immunoprecipitation
PBS: phosphate buffered saline
PTM: post-translational modifications
S-CMC: carbocisteine
SUMO: small ubiquitin-related modifier
TNF-α: tumor necrosis factor alpha.

## References

[B1] Adenuga D. Yao H. March T. H. Seagrave J. Rahman I. (2009). Histone deacetylase 2 is phosphorylated, ubiquitinated, and degraded by cigarette smoke. *Am. J. Respir. Cell. Mol. Biol.* 40 464–473. 10.1165/rcmb.2008-0255OC 18927347 PMC2660563

[B2] Bahnassy S. Kumar S. Ren J. Frutiz G. Karami S. Bawa-Khalfe T. (2017). Androgen receptor in tamoxifen-resistant breast cancer is affected by SUMO. *Cancer. Res* 77(4 Suppl.):P3-04–21.

[B3] Banks R. E. Dunn M. J. Hochstrasser D. F. Sanchez J. C. Blackstock W. Pappin D. J. (2000). Proteomics: new perspectives, new biomedical opportunities. *Lancet* 356 1749–1756. 10.1016/S0140-6736(00)03214-1 11095271

[B4] Barnes P. J. (2005). Targeting histone deacetylase 2 in chronic ovstructive pulmonary disease treatment. *Expert. Opin. Ther. Targets.* 9 1111–1121. 10.1517/14728222.9.6.1111 16300464

[B5] Barnes P. J. (2009). Role of HDAC2 in the pathophysiology of COPD. *Annu. Rev. Physiol.* 71 451–464. 10.1146/annurev.physiol.010908.163257 18817512

[B6] Bossis G. Melchior F. (2006). Regulation of SUMOylation by reversible oxidation of SUMO conjugating enzymes. *Mol. Cell.* 21 349–357. 10.1016/j.molcel.2005.12.019 16455490

[B7] Brandl A. Heinzel T. Krämer O. H. (2009). Histone deacetylases: salesmen and customers in the post-translational modification market. *Biol. Cell.* 101 193–205. 10.1042/BC20080158 19207105

[B8] Brandl A. Wagner T. Uhlig K. M. Knauer S. K. Stauber R. H. Melchior F. (2012). Dynamicallyregulated sumoylation of HDAC2 controls p53 deacetylationand restricts apoptosis following genotoxic stress. *J. Mol. Cell. Biol.* 4 284–293. 10.1093/jmcb/mjs013 22493095

[B9] Citro S. Jaffray E. Hay R. T. Seiser C. Chiocca S. (2013). A role for paralog-specific sumoylation in histone deacetylase 1 stability. *J. Mol. Cell. Biol.* 5 416–427. 10.1093/jmcb/mjt032 24068740

[B10] David G. Neptune M. A. DePinho R. A. (2002). SUMO-1 modification of histone deacetylase 1 (HDAC1) modulates its biologicalactivities. *J. Biol. Chem.* 277 23658–23663. 10.1074/jbc.M203690200 11960997

[B11] Eom G. H. Kook H. (2014). Posttranslational modifications of histone deacetylases: implications for cardiovascular diseases. *Pharmacol. Ther.* 143 168–180. 10.1016/j.pharmthera.2014.02.012 24594235

[B12] Flotho A. Melchior F. (2013). Sumoylation: a regulatory protein modification in health and disease. *Annu. Rev. Biochem.* 82 357–385. 10.1146/annurev-biochem-061909-093311 23746258

[B13] Guizzardi F. Rodighiero S. Binelli A. Saino S. Bononi E. Dossena S. (2006). S-CMC-Lys-dependent stimulation of electrogenic glutathione secretion by humanrespiratory epithelium. *J. Mol. Med.* 84 97–107. 10.1007/s00109-005-0720-y 16283140

[B14] Huang T. T. Wuerzberger-Davis S. M. Wu Z. H. Miyamoto S. (2003). Sequential modification of NEMO/IKKgamma by SUMO-1 and ubiquitin mediates NF-kappaB activation by genotoxic stress. *Cell* 115 565–576. 10.1016/S0092-8674(03)00895-X 14651848

[B15] Ito K. Ito M. Elliott W. M. Cosio B. Caramori G. Kon O. M. (2005). Decreased histone deacetylase activity in chronic obstructive pulmonary disease. *N. Engl. J. Med.* 352 1967–1976. 10.1056/NEJMoa041892 15888697

[B16] Johnson E. S. (2004). Protein modification by SUMO. *Annu. Rev. Biochem.* 73 355–382. 10.1146/annurev.biochem.73.011303.074118 15189146

[B17] Lee J. E. Kim J. H. (2015). SUMO modification regulates the protein stability of NDRG1. *Biochem. Biophys. Res. Commun.* 459 161–165. 10.1016/j.bbrc.2015.02.090 25712528

[B18] Lomelí H. Vázquez M. (2011). Emerging roles of the SUMO pathway in development. *Cell. Mol. Life. Sci.* 68 4045–4064. 10.1007/s00018-011-0792-5 21892772 PMC11115048

[B19] Malhotra D. Thimmulappa R. K. Mercado N. Ito K. Kombairaju P. Kumar S. (2011). Denitrosylation of HDAC2 by targeting Nrf2 restores glucocorticosteroid sensitivity in macrophages from COPD patients. *J. Clin. Invest.* 121 4289–4302. 10.1172/JCI45144 22005302 PMC3204828

[B20] Mann M. Jensen O. N. (2003). Proteomic analysis of post-translational modifications. *Nat. Biotechnol.* 21 255–261. 10.1038/nbt0303-255 12610572

[B21] Mei L. Yuan L. Shi W. Fan S. Tang C. Fan X. (2017). SUMOylation of large tumor suppressor 1 at Lys751 attenuates its kinase activity and tumor-suppressor functions. *Cancer Lett.* 386 1–11. 10.1016/j.canlet.2016.11.009 27847303

[B22] Nott A. Watson P. M. Robinson J. D. Crepaldi L. Riccio A. (2008). S-Nitrosylation of histone deacetylase 2 induces chromatin remodelling in neurons. *Nature* 455 411–415. 10.1038/nature07238 18754010

[B23] Osoata G. O. Yamamura S. Ito M. Vuppusetty C. Adcock I. M. Barnes P. J. (2009). Nitration of distinct tyrosine residues causes inactivation of histone deacetylase 2. *Biochem. Biophys. Res. Commun.* 384 366–371. 10.1016/j.bbrc.2009.04.128 19410558

[B24] Segré C. V. Chiocca S. (2011). Regulating the regulators: the post-translational code of class I HDAC1 and HDAC2. *J. Biomed. Biotechnol.* 2011:690848. 10.1155/2011/690848 21197454 PMC3004424

[B25] Song Y. Lu H. Z. Xu J. R. Wang X. L. Zhou W. Hou L. N. (2015). Carbocysteine restores steroid sensitivity by targeting histone deacetylase 2 in a thiol/GSH-dependent manner. *Pharmacol. Res.* 91 88–98. 10.1016/j.phrs.2014.12.002 25500537

[B26] Tatham M. H. Jaffray E. Vaughan O. A. Desterro J. M. Botting C. H. Naismith J. H. (2001). Polymeric chains of SUMO-2 and SUMO-3 are conjugated to protein substrates by SAE1/SAE2 and Ubc9. *J. Biol. Chem.* 276 35368–35374. 10.1074/jbc.M104214200 11451954

[B27] Teng S. Luo H. Wang L. (2012). Predicting protein sumoylation sites from sequence features. *Amino Acids* 43 447–455. 10.1007/s00726-011-1100-2 21986959

[B28] Wagner T. Godmann M. Heinzel T. (2017). Analysis of histone deacetylases sumoylation by immunoprecipitation techniques. *Methods. Mol. Biol.* 1510 339–351. 10.1007/978-1-4939-6527-4_25 27761833

[B29] Wagner T. Kiweler N. Wolff K. Knauer S. K. Brandl A. Hemmerich P. (2015). Sumoylation of HDAC2 promotes NF-κB-dependent gene expression. *Oncotarget* 6 7123–7135. 10.18632/oncotarget.3344 25704882 PMC4466673

[B30] Woodsmith J. Kamburov A. Stelzl U. (2013). Dual coordination of post translational modifications in human protein networks. *PLoS. Comput. Biol.* 9:e1002933. 10.1371/journal.pcbi.1002933 23505349 PMC3591266

[B31] Xue Y. Zhou F. Fu C. Xu Y. Yao X. (2006). SUMOsp: a web server for sumoylation site prediction. *Nucleic. Acids. Res.* 34 W254–W257. 10.1093/nar/gkl207 16845005 PMC1538802

[B32] Yang M. Hsu C. T. Ting C. Y. Liu L. F. Hwang J. (2006). Assembly of a polymeric chain of SUMO1 on human topoisomerase I in vitro. *J. Biol. Chem.* 281 8264–8274. 10.1074/jbc.M510364200 16428803

